# Predicting net energy in broiler chickens using the comparative slaughter technique

**DOI:** 10.1016/j.psj.2025.105569

**Published:** 2025-07-21

**Authors:** Juan Elmer Moscoso-Muñoz, Liz Beatriz Chino-Velasquez, Jesús Camero de la Cuba, Gardenia Tupayachi Solórzano, Andrés Corsino Estrada Zúñiga, Mario Arjona-Smith, Medardo Antonio Díaz-Céspedes, Oscar Gómez-Quispe, Victor Guevara-Carrasco

**Affiliations:** a Universidad Nacional Agraria La Molina, Lima, Peru; b Universidad Nacional de San Antonio Abad del Cusco, Cusco, Peru; c Universidad de Panama, Panama, Panama; d Universidad Nacional Agraria de la Selva, Tingo Maria, Peru; e Universidad Nacional Micaela Bastidas de Apurímac, Apurimac, Peru

**Keywords:** Net energy for maintenance, Retained energy, Heat increment, Heat production, Comparative slaughter

## Abstract

Two experiments were conducted to determine a prediction model of the net energy (NE) requirement from the net energy for maintenance (NEm) and the retained energy (RE) by the comparative slaughter technique and its validation, and the metabolizable energy was determined using the nitrogen-corrected total excreta collection method. A completely randomized block design was used, and in the validation, the error (%) and the analysis of the concordance correlation coefficient (CCC) were determined. In each experiment, two replicates were used (five chickens per replicate) for each treatment. The experiment included 370 Cobb 500 male chickens (one day old), distributed in two experiments (190 in the first and 180 in the second), fed with six diets, considering a basal diet (corn + soybean meal), from which was substituted on a percentage basis (weight/weight) for the ingredients frequently used in chicken feed (corn, wheat by-product, soybean meal, fish meal and soybean oil) with three feeding levels: *ad libitum* (AL), 85 % (AL) and 70 % (AL) in both experiments. To determine NEm, the relationship between RE and metabolizable energy intake (MEI) was established using simple linear regression. The second study estimated and validated the NE with the proposed model, where the NEm was calculated with simultaneous equations obtained from the basal diet of Study 1. The values of NEm/W^0.75^, EMm/W^0.75^, and the efficiency k, showed similar values among diets (p<0.05). The MEI was greater in the diet with soybean oil than others, the RE represented 45.6 % of the metabolizable energy (ME) consumed and was the greatest in the diet with soybean oil (p<0.05), the heat production on average was 104.59 kcal/day (54.35 % of the MEI), and NEm (kcal/day) was the highest in the diet with soybean oil and represented 24.90 % of the MEI, the heat increment was similar in all diets (29.45 % of the MEI). The estimated NE was similar (141.39 ± 21.13) to the observed NE (137.07 ± 22.28) (p>0.05), with an adequate linear relationship (R^2^ = 0.996) and low error (4.01 %), the CCC was high (0.993) and a υ of 1.02. The proposed net energy model ($NE\;\left(\text{kcal}/\text{day}\right)=79.66\cdot W^{0.75}+RE$), allows to estimate with precision and accuracy the NE requirement in broiler chickens.

## Introduction

It is assumed that net energy (**NE**) represents the most accurate way to value a feed for productive purposes (Barzegar et al., 2020; Liu et al., 2017; Wu et al., 2019). This explains the variations in the metabolic utilization of metabolizable energy (**ME**) from nutrients and ingredients (Noblet, 2015), explains the loss of energy as heat (Swick et al., 2013), and allows for more accurate prediction of the productive response of animals. The composition of diets (type of ingredients and nutrients) influences the efficiency of energy use (heat production), with some ingredients or nutrients being underestimating (fats or starches) or overestimated (protein and fiber) in terms of their true contribution (Barzegar et al., 2020; Morgan et al., 2019; Van der Klein et al., 2020).

The NE is partitioned into net energy for maintenance (**NEm**) and production (**NEp**), therefore, determining the NE of a feed will be influenced by the evaluation of the NEm (Liu et al., 2014; Liu et al., 2017; Zuidhof, 2019). This NEm is affected by animal and environmental factors (Van der Klein et al., 2020). Unfortunately, NEm cannot be determined directly and is difficult to separate from heat production (Zuidhof, 2019). The basal metabolic rate has traditionally been employed to estimate NEm (Noblet et al., 2015), a method affected by fasting time (Ning et al., 2014), previous feeding levels, and differences in activity between fasting and feeding state (Hu et al., 2012; Labussière et al., 2011; Liu et al., 2017). Basal metabolic rate also cannot be measured directly in experiments and is usually estimated by one or more measurements of fasting heat output (Noblet et al., 2015).

The widely used approach to estimate NEm is the regression method (with linear or non-linear models), in which the basal metabolic rate is calculated by extrapolating heat production, measured at different metabolizable energy (ME) consumptions, to zero ME consumption. Non-linear regression models use exponential regression of heat production as a function of a wide range of ME consumption, both above and below maintenance requirements (De Lange and Birkett, 2005). The production NE, also known as retained energy (**RE**), represents the NE supplied beyond maintenance needs and that is used for growth and production (Gutierrez and Patience, 2012).

Determination of NE is expensive and complex, and the method used, such as the comparative slaughter technique, allow knowing the retained energy (protein and fat) and require estimating heat production (Gonçalves et al., 2018; Sakomura et al., 2003; Van der Klein et al., 2020). Indirect calorimetry provides a direct estimate of heat production (Liu et al., 2015; Liu et al., 2016; Li et al., 2017; Yakui et al., 2018) and does not require animals sacrifice, so it has been widely used to determine heat production (Caldas, 2015; Liu et al., 2017). Various prediction equations have been developed using indirect calorimetry as a reference (Liu et al., 2017).

The objective of the NE system cannot be fully achieved because the net energy equations do not take into account variations in factors such as body weight or the physiological state of the animal (Birkett and De Lange, 2001). This leads to the limited use of different net energy values for the same ingredients depending on the type of production (maintenance only or growing) (Ning et al., 2014).

Several equations have been developed to predict the NE of a feed or diet. Depending on the equation, it is necessary to know the nutrients or digestible nutrients in the feed, as inputs to the equation, with or without determining the ME values, making corrections according to their efficiency and for which they are used (Pirgozliev and Rose, 1999). Most of these equations have been developed in experiments using indirect calorimetry (Barekatain et al., 2014; Liu et al., 2017; Li et al., 2017; Rozeboom and Beaulieu, 2011); also NE values of different feeds, such as those measured by Fraps (1946), have been compared with values calculated from different NE prediction equations (Pirgozliev and Rose, 1999). In this context, there is a need to develop a system that allows efficiently predicting of NE values for different ingredients in animal production (Yaghobfar, 2016). To overcome this constraint, we propose developing simulation models that enable a comprehensive characterization of the system while simplifying its estimation. Therefore, the aim of this study was to determine a predictive model of NE requirements from NEm and RE by the comparative slaughter technique and its validation.

## Materials and methods

The approach to the study and use of the animals was approved by the graduate school of Universidad Nacional Agraria La Molina (Lima – Perú).

### Study development

Two experiments were carried out under similar conditions. The first experiment was designed to develop the NEm and the efficiency of utilization of metabolizable energy. In the second experiment, the proposed model was validated to estimate NE from the NEm and the RE.

### Birds and housing

Three hundred and seventy one-day-old Cobb 500 male chickens were used; four battery cages were used (1.20 × 0.60 × 0.20 m, five birds/pen) with five floors or levels each (four divisions per level), with treatments assigned to them randomly. Each level provided with feeders and waterers; the mesh floor and trays for the collection of excreta. Each battery had a thermostat to regulate the temperature according to the aging specifications of the commercial line, the lighting was permanent for 24 hours (artificial lighting). The cages were kept inside a temperature and ventilation controlled building.

### Treatments

In each experiment, the treatments were composed of six experimental diets (Moscoso-Muñoz et al., 2020), considering a basal diet (corn + soybean meal, Control) (Table 1), from which the weight/weight percentage substitution was made for the ingredients frequently used in chicken feed (energy 40 %: corn and wheat by-product, protein 30 %: soybean meal and fish meal, lipid 10 %: soybean oil; Table 2) (Alvarenga et al., 2015; Liu et al., 2016), which were supplied in three feeding levels: *ad libitum* (**AL**), 85 % (AL) and 70 % (AL) (Liu et al., 2017). The chickens received the basal diet until they were seven days of age, after which they were given the experimental diets until they were 21 days of age. The water supply was *ad libitum*, and the feed was based on the proposed restriction levels and each diet was provided in mash form.

**Table 1:** Nutritional composition calculated of the basal diet (as fed).

Ingredient	Amount (%)
Corn	62.73
Soybean meal (44 % CP)	31.39
Soybean oil	1.69
Dicalcium Phosphate	2.11
Calcium carbonate	0.93
Salt	0.48
DL - Methionine	0.31
L - Lysine HCL	0.21
Premix ^a^	0.10
Calculated composition	
Metabolizable energy (Mcal/kg)	3.07
Crude protein (%)	20.49
Lysine (%)	1.26
Arginine (%)	1.36
Methionine (%)	0.61
Methionine + Cystine (%)	0.94
Available phosphorus (%)	0.45
Calcium (%)	0.90
Sodium (%)	0.20

Premix of vitamins and minerals per kilogram of fed: Vitamin A 9000 IU, Vitamin D_3_ 2000 IU, Vitamin E 16.0 IU, Vitamin K 2.0 mg, Riboflavin 5.5 mg, Niacin 53.0 mg, Calcium D-Pantothenate 11.0 mg, Folic Acid 0.1 mg, BHT. 100.0 mg, Manganese 112.0 mg, Zinc 100.0 mg, Iron 56.0 mg, Copper 7.0 mg, Iodine 1.0 mg, Selenium 0.1 mg.

**Table 2:** Diets used in the study by substitution level and their determined nutritional value ( %, dry basis).

	Basal (B)	B+Corn	B+WBP	B+Soybean meal	B+ Fish meal	B+Oil (soybean)
Substitution level	-	40	40	30	30	10
Bassal diet	100	60	60	70	70	90
Total	100	100	100	100	100	100
Moisture, %	11.38	11.70	11.51	11.14	10.28	10.23
Crude protein, %	23.57	17.70	19.91	31.67	38.33	21.13
Ether extract, %	3.45	2.77	3.99	3.17	5.49	11.61
Crude fiber, %	2.73	2.39	5.50	3.42	2.04	2.65
Nitrogen-free extract, %	63.51	72.27	64.33	54.73	43.90	58.58
NDF, %	10.17	10.17	23.75	10.42	16.19	11.16
Ash, %	6.74	4.87	6.26	7.01	10.23	6.03
GE, kcal/kg	4444	4356	4491	4437	4451	4928

Abbreviations: GE: gross energy (bomb calorimeter); NDF: neutral detergent fiber; B+WBP: Basal+wheat by-product.

Mixing proportions between ingredients and basal diet are expressed on a fresh basis.

The excreta were collected at 24-hour intervals on three successive days during the last week of the experimental period (day 19 to 21). Once collected, they were cleaned, weighed and frozen at -20°C for later analysis.

Experiment 1 Net Energy content and construction of the model to estimate the net energy.

The NE for each diet, was determined by the comparative slaughter tecnhique (Sakomura and Rostagno, 2017); 180 one-day-old Cobb 500 male chickens randomly distributed were used (six diets, three feeding levels, two repetitions and five chickens per repetition). Additionally, at the beginning of the evaluation (7 days) 10 chickens were sacrificed by cervical dislocation without blood loss (Gous, 2010) to determine the RE (making a total of 190 chickens in the first experiment). The animals were weighed at reception (one day of age), at seven and 21 days, feed intake was recorded in these periods. Once the experiment was completed, the chickens were sacrificed following the same procedure, in both cases the sacrifice was made after 8 hours of fasting, then the chickens (including carcass, feathers and viscera) were frozen (-20°C) and stored for later analysis.

Frozen chickens were first cut into small pieces, then mixed and ground with a meat grinder. Weighing was carried out on a precision electronic balance before and after drying to calculate dry matter (**DM**) and develop subsequent analyses.

Body weight gain (**BWG**) and average weight (**W**) were calculated as follows:

$$BWG,\;g=\frac{Weight_{21\;day}-Weight_{7\;day}}{14\;day}$$

$$W,\;g=\frac{Weight_{21\;day}-Weight_{7\;day}}{2}$$

Feed intake (**FI**):

$$FI,\;g\;DM/day=\frac{FI_{21\;day}-FI_{7\;day}}{14\;day}$$

Metabolizable energy intake (**MEI**): The ME was determined by the nitrogen-corrected total excreta collection method (Wu et al., 2019).

$$MEI\;\left(\text{kcal}/\text{day}\right)=ME_{\text{diet}}\;\left(\text{kcal}/g\;\text{DM}\right)\cdot FI\;\left(g/\text{day}\right)$$

Metabolic body weight (W^0.75^): This measure was employed because it serves as a standardized metric in animal nutrition, accounting for the nonlinear relationship between body weight (BW) and metabolic rate. In the study, metabolic body weight was calculated as BW raised to the exponent 0.75 (BW⁰·⁷⁵).

$$W^{0.75}={\left(BW\right)}^{0.75}$$

***Retained energy.*** The total body energy at the beginning of the experiment was determined as the value of total body energy present in all experimental groups. Then the final body energy value of the chickens at the end of the experiment was determined, whose values were multiplied by the individual weight gains.

$RE,\;kcal=RE_{21\;day}-RE_{7\;day}$, where RE is the retained energy.

$$RE,\;kcal/day=\frac{RE_{21\;day}-RE_{7\;day}}{14\;day}$$

The NE, heat production (**HP**), and heat increment (**HI**) of the diet were calculated with the following equations:

NE(kcal)=k·ME, kcal/kg; where **k** is the net energy use efficiency for maintenance.

The value of k was obtained from a simple linear regression between metabolizable energy and retained energy (both expressed in metabolic units) and k represents the slope of the regression line.

HP,kcal/day=MEI(kcal/day)−RE(kcal/day)

HI=HP−NEm

Therefore, NEm and NE were calculated with the following equations:

NEm,kcal/day=HP(kcal/day)−HI(kcal/day)

and

NE,kcal/day=NEm(kcal/day)+RE(kcal/day)

Experiment 2 ***Validation of the model to estimate the Net Energy requirement***

In the second experiment, the validation of the net energy requirements was carried out with the proposed model.

***Estimation and validation model of Net Energy requirements.*** The net energy requirements were estimated based on the following model:

$$NE=a\cdot W^{0.75}+RE$$

With the basal diet of Experiment 1, the coefficient “a” that precedes the metabolic weight (W^0.75^) was calculated to determine the NEm, for this simultaneous equations were used with the modified Fraps (1946) technique and, the retained energy values obtained in the second study, the same as presented below:

$$\mathrm{RE}/W^{0.75}=-a\;+\;\mathrm{kME}/W^{0.75}\;\;\;\;\;\;\;\;\;\;\;\;\;\;\;\;\;\;\;\;\;\;\;\;\;\;\;\;\;\;\;\;\;\;\;\;\;\;\;\;\;\;\mathrm{Ad}\;\mathrm{lib}$$

$$\mathrm{RE}/W^{0.75}=\;-a\;+\;\mathrm{kME}/W^{0.75}\;\;\;\;\;\;\;\;\;\;\;\;\;\;\;\;\;\;\;\;\;\;\;\;\;\;\;\;\;\;\;\;\;\;\;\;\;\;\;\;\;\;70\%\;\mathrm{AL}$$

Retained energy, was obtained from the analysis of body composition.

The validation of the model was carried out with the values determined in the second study, in which the same experimental conditions, and types of diets were used as those used in study 1.

### Sample analysis

The chemical analysis of the diets, ingredients, chickens, and excreta were taken to the Nutrition, Food Science and Technology Laboratory of the Universidad Nacional de San Antonio Abad del Cusco, the Nutritional Food Evaluation Laboratory of the Universidad Nacional Agraria La Molina and the Laboratory of Biochemistry, Nutrition and Animal Feed of the Universidad Nacional Mayor de San Marcos for analysis according to the methodology proposed by the Association of Analytical Chemists (AOAC). Dry matter content was determined in a forced air circulation oven (FED 720, Binder GmBH, Tuttlingen, Germany) at 135°C for 2 h (method 930.15; AOAC, 2005). Nitrogen was analized using elemental analyzer (2400 Series II, PerkinElmer Inc., Waltham, MA, USA) (method 990.03; AOAC 2005), and crude protein was calculated by multiplying the percentage of N by a correction factor (6.25). Ether extract was analized using an automatic crude fat analyzer (SOX 606, Hanon Instruments Co., Ltd., Jinan, China) (method 2003.05; AOAC 2005). Crude fiber was analized using an automatic crude fiber analyzer (Fibretherm FT12, C. Gerhardt GmbH & Co. KG, Königswinter, Germany) (method 962.09, AOAC, 2005). Ash content was determined by incineration samples in a muffle furnace (ECO110/9, Protherm Furnaces, Ankara, Turkey) at 600°C for 2 h (method 942.05; AOAC, 2005). Neutral detergent fiber (NDF) was determined using an automated fiber analysis system (Fibretherm FT12, C. Gerhardt GmbH & Co. KG, Königswinter, Germany) (Table 2).

The chickens were dried in a forced air circulation oven (FED 720, Binder GmbH, Tuttlingen, Germany) at 55°C for 96 hours and ground at 1 mm (MF10 Basic, IKA-Werke GmbH & Co. KG, Staufen, Germany) to obtain adequate homogenization (Rochell and Dozier, 2011). The gross energy of the diets, ingredients, chickens (retained energy), and excreta was determined in an automatic isoperibol bomb calorimeter (6400 Calorimeter, Parr Instrument Company, Moline, IL, USA) using benzoic acid as a standard. Samples were weighed on an analytical balance (200 ± 0.01 g; Quintix 224-1X, Sartorius AG, Göttingen, Germany) and an ultra-microbalance (5 g/0.1 µg; AD 6000, PerkinElmer Inc., Waltham, MA, USA). Nitrogen, ether extract, ash, and dry matter were analyzed in both chickens and excreta.

### Statistical analyses

The study was carried out under a complete randomized block design, the comparison between treatment averages was determined using the Tukey test (5 %). Firstly, the normal distribution (Anderson and Darling tests) and homogeneity of variance (Levene's test) were verified. The data analysis were performed using the Jamovi 2.4 software. To determine the percentage of error, the comparison between the determined and estimated values of the NE was used, first calculating the mean square of the predicted error and percentage of predicted error, expressed as follows (Ellis et. al., 2010; Sales, 2009):

$$MSPE=\frac{1}{n}\sum_{i=1}^{n}{\left(O_{i}-P_{i}\right)}^{2}$$

$$\%e=\sqrt{MSPE}.\left(\frac{100}{\overset{¯}{y}}\right)$$

Where MSPE is the mean square of the predicted error, n is the total number of observations, Oi is the observed value, Pi is the predicted value, ŷ is the average of the observed values and % e is the percentage of predicted error.

The error (RMSPE) was decomposed into error due to overall bias (ECT), error due to deviation from the slope of the regression from unity (ER), and error due to disturbance (ED) known as random error (Ellis et al., 2010; Velarde-Guillén et al., 2019). These were calculated as follows:

$$\mathrm{ECT}={\left(P_{j}-O_{i}\right)}^{2}$$

$$\mathrm{ER}={\left(S_{p}-R\cdot S_{o}\right)}^{2}$$

$$\mathrm{ED}=\left(1-R^{2}\right)\cdot S_{o}^{2}$$

Where **S_p_** is the predicted standard deviation, **R** is the Pearson correlation coefficient and **S_o_** is the observed standard deviation.

The analysis of the concordance correlation coefficient was also performed (**CCC**) (Lin, 1989), which was calculated as follows:

$$CCC=R\cdot C_{b}$$

Where C_b_ is a bias correction factor. The R variable gives a measure of precision and C_b_ is a measure of accuracy; The C_b_ variable is calculated as:

$$C_{b}=\frac{2}{\nu +\frac{1}{\nu }+\mu^{2}}$$

$\nu =\frac{S_{o}}{S_{p}}$
  $$\mu =\frac{O_{i}-P_{j}}{\sqrt{S_{o}\cdot S_{p}}}$$

**Where υ provides a measure of scale shift (change in standard deviation between predicted and observed values), while µ provides a measure of location shift.**

## Results and discussion

### Net Energy for maintenance and efficiency of utilization of metabolizable energy

The results of the regression analysis between retained energy (RE) and metabolizable energy intake (MEI) are presented in Table 3. NEm values varied from 62 to 91 kcal NEm/W^0.75^/day but did not show significant differences among diets. Liu et al. (2017) determined values of 92 kcal NEm/W^0.75^/day (indirect calorimetry) and 97 kcal NEm/W^0.75^/day through comparative slaughter. Sakomura et al. (2005) using comparative slaughter, established values of 119.30, 89.99, and 96.25 kcal NEm/W^0.75^/day, for temperatures of 13, 23, and 32°C respectively. In general, the differences observed in the present study, when compared with other authors, would be determined by the genotype of the chickens used in the experiments, and environmental rearing conditions (Liu et al., 2017; Sakomura et al., 2003; Shatnawi, 2014; Van der Klein et al., 2020), method used for the determination (Liu et al., 2017) and the composition of the carcass (Sakomura et al., 2005).

**Table 3:** Linear regression analysis of retained energy (RE) as a function of metabolizable energy intake (MEI).

Diet	Regression equation	NEm (kcal/W^0.75^/day)	MEm (kcal/W^0.75^/day)	k (%)	R^2^
Basal (B)	RE=−75.28+0.71·MEI	75.28	106.68	0.71	0.85
B + corn	RE=−85.55+0.68·MEI	85.55	125.02	0.68	0.99
B + wheat by-product	RE=−91.31+0.69·MEI	91.31	132.75	0.69	0.83
B + soybean meal	RE=−75.89+0.71·MEI	75.89	107.03	0.71	0.94
B + fish meal	RE=−62.43+0.65·MEI	62.43	96.68	0.65	0.93
B + soybean oil	RE=−83.02+0.73·MEI	83.02	114.34	0.73	0.93
Average		79.64	113.75	0.70	
P-value		0.19	0.14	0.10	

NEm: net energy requirement for maintenance, MEm: metabolizable energy for maintenance, k: net energy use efficiency for maintenance. W^0.75^: metabolic body weight. RE: retained energy. $MEm=\frac{NEm}{k}$.

The efficiency of the use of metabolizable energy was similar in all diets, ranging of 65 % to 73 %, while the efficiency values ​​determined by Liu et al. (2017) were 65 % (indirect calorimetry) and 65.40 % (comparative slaughter), values ​​similar to those obtained in the present study by linear regression. When the calculation is considered using logarithmic regression, the efficiencies are higher (with values ​​of 73.8 % by indirect calorimetry and 75 % by comparative slaughter). Sakomura et al. (2005), using logarithmic regression, established efficiency values ​​of 76 % and 80 %, with lower efficiency in birds raised at low temperatures, coinciding with the results of the present study.

Metabolizable energy for maintenance (MEm) was similar in all diets with values ranging between 96.68 kcal MEm/W^0.75^/day and 132.75 kcal MEm/W^0.75^/day (Table 3). These results are lower than those reported by Liu et al. (2017) who estimated 141.85 and 147.58 kcal MEm/W^0.75^/day by indirect calorimetry and by comparative slaughter respectively. For their part, Nieto et al. (1995) reported values from 110.8 to 142.65 kcal MEm/W^0.75^/day, alleging that the variation of these values is a function of the biological value of dietary protein, having an inverse relationship with MEm. Sakomura et al. (2005) determined that MEm was 157.8, 112.1, and 127.2 kcal MEm/W^0.75^/day for chickens raised at 13, 23, and 32°C, respectively. Noblet, et al. (2015) established a broader range of MEm from 81 to 187 kcal, within which the values reported in the present study are found.

The results show variations in NEm, MEm and **k** efficiency, depending on the type of diet consumed by the chickens (Shatnawi, 2014). These differences could be primarily attributed to variations in the nutritional composition of the ingredients (Noblet et al., 1999), the presence of antinutritional factors that impair energy utilization (García-Rebollar et al., 2016). This leads to variations in nutrient utilization efficiency.

### The energy utilization

MEI was higher in the diet with soybean oil, followed by the diets with corn and wheat by-products, and lower in the basal diet, soybean meal, and fish meal (p<0.001; Table 4). The RE, on average, was 88.65 kcal/day and represented 45.6 %, with a range of 42.1 to 51.3 % of the ME consumed (Table 4), which is within the ranges recorded in the literature (Shatnawi, 2014), being higher in the diet with soybean oil followed by the diet with corn, but lower in the diets with fish meal, soybean meal, wheat by-product and basal (p<0.01).

**Table 4:** Energy utilization in experimental diets in experiment I.

	Basal (B)	B+Corn	B+WBP	B+Soybean meal	B+Fish meal	B+Oil (soybean)	P-value
BW 21 day, kg	0.813^c^	0.798^c^	0.796^c^	0.876^bc^	0.964^ab^	0.969^a^	<0.001
BW 7 day, kg	0.158	0.163	0.171	0.161	0.156	0.166	0.537
Average BW, kg	0.486^c^	0.480^c^	0.484^c^	0.518^b^	0.560^a^	0.568^a^	<0.001
W^0.75^	0.582^a^	0.577^a^	0.580^a^	0.611^b^	0.647^a^	0.654^a^	<0.001
ADG, g/day	46.80^c^	45.32^d^	44.66^d^	51.08^b^	57.73^a^	57.36^a^	<0.001
ME intake, kcal/day	174.47^c^	210.48^b^	184.58^bc^	171.40^c^	183.85^c^	234.63^a^	<0.001
NE, kcal/day	127.24^cd^	145.01^b^	130.87^c^	123.59^cd^	117.71^d^	174.66^a^	<0.001
RE, kcal/day	83.44^c^	95.64^b^	77.93^c^	77.22^c^	77.31^c^	120.35^a^	<0.001
NEm, kcal/day	43.80^e^	49.36^c^	52.95^b^	46.37^d^	40.40^f^	54.31^a^	<0.001
HP, kcal/day	91.03^b^	114.83^ab^	106.66^a^	94.17^ab^	106.54^ab^	114.28^a^	0.017
HI, kcal/day	47.23	65.47	53.71	47.80	66.14	59.97	0.055

BW: body weight; ADG: average daily gain; RE: retained energy; NE: net energy; ENm: net energy requirement for maintenance; RE: retained energy; HP: heat production; HI: heat increment; W^0.75^: metabolic body weight; B+WBP: basal+wheat by-product; ME: metabolizable energy.

Shatnawi (2014) found that diets with oil (B+soybean oil) and starch (B+corn) have greater energy retention compared to diets with casein (higher protein), determined by the reduction in caloric increase (Shannon and Brown, 1969) since this is lower when ME is used for fat deposition compared to protein deposition, the cost of which is high (Noblet et al., 1999). This fact implies that the differentiated responses among types of diets are determined by the efficiency of the nutrients used (Carré et al., 2014; Cerrate-Fernandez et al., 2019; Swick et al., 2013). When the energy: protein ratio is low, it implies high levels of protein in the diet (B+fish meal and B+soybean meal), generating a high caloric increase, because protein synthesis requires a large amount of energy, energy is also required to excrete nitrogenous waste and, in addition, the dietary protein stimulates protein turnover (Gous et al., 2018; Musharaf and Latshaw, 1999).

Different studies show that the RE derived from ingredients high in protein, such as those from animal sources, and fiber, were lower than those derived from ingredients high in starch (cereals), attributing it to the greater heat production (Jørgensen et al., 1996) and energy loss (Choct, 1999). It was associated with low metabolic efficiency (Noblet et al. 1994). On the other hand, ingredients from feeds rich in fat have a high NE value (Shatnawi, 2014) and a low caloric increase (Shannon and Brown, 1969; Sibbald, 1982). Diets with high fiber levels (B+wheat by-product) increase the volume of excreta, determining an increase in the size of the gastrointestinal tract, mainly the weight of the cecum, also decreasing the digestibility of nutrients (Jørgensen et al., 1996).

In the present study, heat production (HP) was 104.59 kcal/day on average, which represented 54.35 % of the ME consumed, it was lower in the basal diet compared to the other diets. Lopez and Leeson (2008) mention that, under thermoneutral conditions, HP represents 52 to 64 % of the ME consumed; variations in HP could be due to many factors such as environmental temperature, as well as the consumption and composition of the feed (Liu et al., 2016), the type of activity carried out by the animals (Musharaf and Latshaw, 1999) and the body composition (Priyankarage et al., 2011).

Considering that HP is a function of NEm and HI, it can be seen that the NEm was higher in the diet with soybean oil, compared to the other diets (p<0.001), and represents 24.90 % of ME intake. One reason such differences can be observed would be the variations in the metabolic weight of the animals (Noblet et al., 2015). On the other hand, the caloric increase observed in the present study was similar in all diets, representing 29.45 % of the consumed ME varying from 25.56 % to 35.98 %. These results agree with Van der Klein et al. (2020) who also found no differences in HI between diets with high and low energy levels (26 % of MEI). Musharaf and Latshaw (1999) stated that the greatest caloric increase determined by dietary protein would be a function of its inclusion level; with standard diets, the caloric increase represents 25 % of the ME intake in growing pigs, chickens, and turkeys (Noblet et al., 1994; Rivera-Torres et al., 2011).

The net energy was on average 136.51 kcal/day, which corresponds to 70.55 % of the ME consumed, varying from 64 - 73 %, which is lower than mentioned by Yadalam (2001) who indicates that the NE represents 84 % of the ME in chickens 0 – 21 days old. In the present study, NE was higher in the diet with soybean oil, followed by the diet with corn, and lower in the other diets (p<0.001). This characteristic would be associated with the extra caloric effect that lipids have, which increases the availability of dietary nutrients (Murugesan and Latshaw, 2013). Wu et al. (2019) mention that NE has a positive relationship with ME and ether extract, but this relationship is negative with crude protein.

These fundings show the effect that the composition of diets (nutrient content) has on net energy and energy retention, since the proportion of protein, fat, or starch in the ingredients affects the efficiency of the use of ME (Pirgozliev and Rose, 1999), generating variations in heat production and caloric increase (Cerrate-Fernandez et al., 2019; Noblet et al., 1999; Swick et al., 2013). Differences in net energy in grains are correlated with differences in the performance of growing chickens, which are not detected by metabolizable energy determinations (Lasek et al., 2020).

### Net energy requirement estimation model and its validation

In the present study, the net energy was estimated from the net energy maintenance obtained in the first experiment based on modified Fraps (1946) (Table 5) and the retained energy values obtained in the second study (Table 6). Validation was performed with the values determined in the second study (Table 7). Thus, the model for net energy maintenance and net energy was:

Basal diet:
  $$\begin{array}{ccc} 299.83X+Y & = & 143.39\;\;\;\;\;\;\;\;\;\;\;\;\;\;\;\;\;\;\;\;\;\;\;\;\;\;\;\;\;\;\;\;\;\;\;\;\;\;\;\;\;\;\mathrm{Ad}\;\mathrm{lib}\\ 231.85X+Y & = & 92.82\;\;\;\;\;\;\;\;\;\;\;\;\;\;\;\;\;\;\;\;\;\;\;\;\;\;\;\;\;\;\;\;\;\;\;\;\;\;\;\;\;\;70\%\;\mathrm{AL}\\ NEm\;\left(\text{kcal}/\text{day}\right) & = & 79.66\cdot W^{0.75}\\ NE\;\left(\text{kcal}/\text{day}\right) & = & 79.66\cdot W^{0.75}+RE\\ k & = & 0.74 \end{array}$$

**Table 5:** Energy utilization considering restriction levels for the construction of the NEm prediction model (basal diet – experiment I).

	100 %	85 %AL	70 %AL
BW 21 day, kg	0.813^c^	0.693	0.574
BW 7 day, kg	0.158	0.168	0.165
Average BW, kg	0.486^c^	0.427	0.369
W^0.75^	0.582^a^	0.529	0.474
ADG, g/day	46.80^c^	37.94	29.25
ME intake, kcal/day	174.47^c^	144.56	109.87
NE, kcal/day	127.24^cd^	95.85	79.66
NEm, kcal/day	43.80^e^	39.80	35.67
HI, kcal/day	47.23	48.71	30.21
HP, kcal/day	91.03^b^	88.51	65.88
RE, kcal/day	83.44^c^	56.05	43.99
REp, kcal/day	46.25	40.29	32.21
REl, kcal/day	37.18	14.82	9.31

BW: body weight; ADG: average daily gain; RE: retained energy; NE: net energy; NEm: net energy requirement for maintenance; RE: retained energy; HP: heat production; HI: heat increment; W^0.75^: metabolic body weight; REp: retained energy as protein; REl: retained energy as lipid; ME: metabolizable energy.

**Table 6:** Net energy determined in experiment II.

Characteristics	Basal (B)	B+Corn	B+WBP	B+Soybean meal	B+ Fish meal	B+Oil (soybean)
Ad libitum feeding						
Average BW, kg	0.539	0.509	0.458	0.520	0.565	0.563
W^0.75^	0.629	0.603	0.557	0.612	0.651	0.650
Feed intake, g MS/day	71.74	74.04	69.28	62.43	62.07	69.54
ME intake, kcal/day	226.51	238.01	201.03	171.12	194.25	258.40
ER, kcal/day	95.79	112.80	72.81	76.36	77.23	118.47
k, determined	73.98	70.19	68.19	74.68	70.48	77.71
NEm, kcal/day	46.54	42.30	37.95	45.73	45.91	50.50
NE, kcal/day	142.33	155.09	110.76	122.09	123.13	168.98

BW: body weight; W^0.75^: metabolic body weight; ME: metabolizable energy; RE: retained energy; NEm: net energy requirement for maintenance; NE: net energy; k: net energy use efficiency for maintenance; B+WBP: basal+wheat by-product.

**Table 7:** Results of the error test and coefficient of determination for the NE of the diets from the proposed model ($NEm=79.66\cdot W^{0.75}+RE$).

Diet	BW, kg		W^0.75^		NEm, kcal/day		RE, kcal/day		NE, kcal/day estimated	NE, kcal/day determined
Ad libitum feeding										
Basal	0.539		0.629		50.12		95.79		145.91	142.33
B + corn	0.509		0.603		48.00		112.80		160.80	155.09
B + wheat by-product	0.458		0.557		44.33		72.81		117.14	110.76
B + soybean meal	0.520		0.612		48.78		76.36		125.14	122.42
B + fish meal	0.565		0.651		51.89		77.23		129.11	123.13
B + soybean oil	0.563		0.650		51.77		118.47		170.24	168.98
Average									141.39	137.07
Standard deviation									21.13	22.28
R										0.998
R^2^										0.996
MSPE										17.68
% error										4.01
ECT, %										82.72
ER, %										0.49
ED, %										16.19
CCC										0.988
C_b_										0.990
υ										1.01
µ										-0.14

BW: body weight; W^0.75^: metabolic body weight; NEm: net energy for maintenance; ER: retained energy; NE: net energy; MSPE: mean square prediction error; R^2^: determination coefficient. Cb: is a bias correction factor; υ: scale shift; µ: location shift; CCC: concordance correlation coefficient analysis; ECT: error due to overall bias; ER: error due to deviation of the regression slope from unity; ED: error due to the disturbance (random error).

When validating the NE based on the proposed model, it is shown that the estimated NE (141.39 ± 21.13 kcal/day) was similar to the observed one (137.07 ± 22.28 kcal/day), with an adequate linear relationship (R^2^ = 0.996) and a low error rate (4.01 %). Most of this error comes from the highest value estimated with the model (ECT = 82.72 %) which averaged 3.85 kcal/day and to a lesser extent from the random effects (ED = 16.19 %); the fraction that corresponds to the error of the regression (regression slope) is minimal (ER = 0.49 %), indicating that the simulation was adequate.

The CCC analysis had a high value (0.988) and a υ close to 1 (1.01), implying a relatively equal standard deviation, the value of negative µ (-0.14) corroborating what was previously indicated, that the values estimated by the model are greater than the determined values, with minimal differences, therefore, the model is valid and predicts adequately with high precision and accuracy (Table 7). Similar results were obtained by Cerrate and Coon (2010) in the evaluation of a biochemical model to estimate the net energy of diets and inputs in chickens, using a theoretical NEm value showing that the energy transformations and losses associated with the use of feed for maintenance and production can be expressed in physiological and biochemical terms.

Liu et al. (2017), applying a linear regression between retained energy and metabolizable energy consumption and also a logarithmic regression equation between heat production and metabolizable energy consumption (using the comparative slaughter technique and indirect calorimetry), also found high determination coefficients between 0.92 (indirect calorimetry) and 0.97 (comparative slaughter) to estimate net energy for maintenance.

The study results demostrate that the use of the comparative slaughter technique allows the determination of retained energy and calculate the heat production through the metabolizable energy intake to retained energy ratio, thus allowing the construction of a prediction model that simplifies the calculation of the coefficient k and the net energy for maintenance. However, further research on this proposed model is necessary, particularly regarding the number of replicates (especially for feed intake calculations) and animal age (evaluations at 14 and 28 days).

## Conclusions

Although some parameters were similar, the soybean oil diet allowed a higher intake, retention and utilization of metabolizable energy in broilers. Furthermore, heat production was lower in the basal diet, while net energy reached its highest values ​​in the soybean oil diet (70.55 % of ME consumed). The proposed net energy model: $NE\;\left(\text{kcal}/\text{day}\right)=79.66\cdot W^{0.75}+RE$, allows us to efficiently estimate the Net Energy requirements in broilers with high precision and accuracy (error of 4.01 % and a correlation coefficient of agreement of 0.988). The results of this study demonstrate that net energy requirements can be estimated using the comparative slaughter technique (to determine retained energy) and simple linear regression, which calculates the k factor and, consequently, the net energy required for maintenance.

## Disclosures

The authors declare that they have no known competing financial interests or personal relationships that could have appeared to influence the work reported in this paper.
